# T2-based temperature monitoring in bone marrow for MR-guided focused ultrasound

**DOI:** 10.1186/s40349-016-0073-8

**Published:** 2016-11-17

**Authors:** Eugene Ozhinsky, Misung Han, Matthew Bucknor, Roland Krug, Viola Rieke

**Affiliations:** Department of Radiology and Biomedical Imaging, University of California San Francisco, 185 Berry Street, Suite 350, Box 0946, San Francisco, CA 94107 USA

**Keywords:** MR-guided focused ultrasound, HIFU, MR thermometry, T2 mapping, Bone marrow

## Abstract

**Background:**

Current clinical protocols for MR-guided focused ultrasound (MRgFUS) treatment of osseous lesions, including painful bone metastases and osteoid osteomas, rely on measurement of the temperature change in adjacent muscle to estimate the temperature of the bone. The goal of this study was to determine if T2-based thermometry could be used to monitor the temperature change in bone marrow during focused ultrasound ablation of bone lesions.

**Methods:**

We investigated the dependence of T2 on temperature in ex vivo bovine yellow bone marrow at 3T and studied the influence of acquisition parameters on the T2 measurements. We examined if T2 changes in red bone marrow caused by the ablation of ex vivo trabecular bone were reversible and measured the patterns of heating and tissue damage. The technique was validated during the ablation of intact ex vivo bone samples and an in vivo animal model.

**Results:**

Results of the calibration experiment showed a linear relationship (7 ms/°C) between T2 change and temperature and could be used to quantify the temperature during heating of up to 60 °C. During trabecular bone ablation, we observed a linear relationship (5.7 ms per °C) between T2 and temperature during the heating stage of the experiment. After cool down, there was residual T2 elevation (~35 ms) in the ablated area suggesting irreversible tissue changes.

In ex vivo and in vivo cortical bone ablation experiments, we observed an increase in T2 values in the marrow adjacent to the intersection of the cortical bone and the beam path. The in vivo experiment showed excellent correspondence between the area of T2 elevation in marrow during the ablation and the resulting non-enhancing area in the post-contrast images.

**Conclusions:**

In this study, we have demonstrated that T2-based thermometry can be used in vivo to measure the heating in the marrow during bone ablation. The ability to monitor the temperature within the bone marrow allowed more complete visualization of the heat distribution into the bone, which is important for local lesion control.

## Background

MR-guided focused ultrasound (MRgFUS) is a noninvasive ablation technique that has grown tremendously over the past years for a broad array of applications such as treatment of uterine fibroids [1], painful bone metastases [2], breast cancer [3], and hepatocellular carcinoma [4] along with thalamotomy for essential tremor [5] and blood-brain barrier manipulation for chemotherapy [6]. Palliative treatment of bone metastases has been Food and Drug Administration (FDA) approved since 2012 and offers a safe and effective noninvasive treatment option for metastatic bone pain, with more than 70 % of patients experiencing fast pain reduction within 3 days after treatment [2, 7, 8]. While the current treatment goal is pain control through periosteal nerve ablation, local tumor control has increasingly become of interest as a primary treatment option [9, 10]. Recent studies have shown that aggressive treatment (increased temperature and duration) is promising for local tumor control [9]. In addition to palliation of painful bone metastases, treatment of osteoid osteomas [11, 12] and facetogenic back pain [13] has also shown significant promise in early clinical studies.

Proton resonant frequency (PRF) shift thermometry is the standard method for monitoring temperature during MRgFUS interventions [14]. It can precisely measure the changes in temperature in water-based tissues, but fails to detect temperature changes in bone and in tissues with high lipid content, such as bone marrow. In water, the dependence of the PRF on temperature is attributed to changes in the hydrogen bonds, which are absent in fat [15]. Therefore, current clinical protocols rely on the measurement of temperature change of the adjacent muscle to estimate the temperature of the bone. This approach carries a significant risk of overtreatment in that more energy might be used than is needed to ablate bone. In fact, we observed in focused ultrasound (FUS) treatments of bone metastases, that the highest temperature in soft tissue was only reached 10–15 s after the ultrasound (US) was switched off [16]. Collateral treatment of the near-field soft tissues during MRgFUS increases the risk for muscle and vascular injury, which can result in significant perioperative or chronic pain.

For local control of osseous lesions, it is often beneficial to achieve deeper penetration of the ablation through the cortical bone into the bone marrow or tumor. In the treatment of osteoid osteomas, complete ablation of the nidus is required for sustained pain relief, but the thickened cortical bone around the nidus makes ultrasound penetration difficult. For such applications, temperature measurement within the bone is critical to reduce the risk of recurrence.

Direct temperature assessment in the bone using MRI has been recently achieved using short and ultrashort echo time (UTE) pulse sequences. Han et al. proposed the use of UTE for quantifying the temperature change in the cortical bone by measuring T1 changes [17]. Ramsay et al. showed a temperature-dependent change in the signal magnitude of the cortical bone at short echo time (1 ms) [18]. However, the applications of these techniques for in vivo monitoring of focused ultrasound therapy are primarily hampered by signal-to-noise ratio (SNR) constraints as well as long acquisition times.

Another way to quantify the effects of heating within the bone would be to measure the temperature in bone marrow. Red bone marrow contains blood forming cells and fat (20–80 % by volume) and is found in flat bones as well as in the trabecular (cancellous) tissue at the epiphyseal ends of long bones [19]. Yellow marrow mainly consists of fat cells (more than 80 % fat by volume) and is found in the medullary cavity of long bones. Relaxation times of red bone marrow have been measured by Schick et al. [20]. Previous studies have shown that a change in T2 can be used to measure the temperature change in other fatty tissues such as subcutaneous fat and breast tissue [21–23]. Heijman et al. have demonstrated the feasibility of T2-based MR thermometry in extracted samples of yellow and red bone marrow [24], but the lack of calibration data for bone marrow at 3T made it difficult to convert T2 values into maps of tissue temperature.

The goal of this study was to determine if T2-based thermometry could be used to monitor the temperature change in ex vivo and in vivo bone marrow during focused ultrasound ablation of bone lesions. We investigated the dependence of T2 on temperature in ex vivo bovine yellow bone marrow at thermal equilibrium at 3T and studied the influence of acquisition parameters on the T2 measurements. We also examined if T2 changes in red bone marrow caused by the ablation of ex vivo trabecular bone were reversible and measured the patterns of heating and tissue damage. Finally, we validated the technique during the ablation of intact ex vivo bone samples and during an in vivo animal experiment.

## Methods

### Yellow bone marrow temperature calibration

Bovine yellow bone marrow was extracted from the medullary cavity of a femur segment, filled into a petri dish, and placed in an insulated cylindrical container. The container was filled with deionized water, which was circulated by a peristaltic pump (Masterflex, Cole Parmer, Vernon Hills, IL) through a heat exchanger. The heat exchanger was placed in a temperature-regulated water bath (Polystat R6L, Cole-Parmer) set to the desired temperature. To reduce heat loss from the heat exchanger in the water bath outside the scanner room to the sample container in the scanner bore, all tubes were covered with pipe insulation. The temperature in the water-filled container was monitored with a fiber optic sensor (Luxtron, LumaSense Technologies, Santa Clara, CA). The time necessary for temperature equilibration within the sample has been determined previously [23] by measuring the time it took to reach equilibrium after a 10 °C change in circulating water temperature which was found to be approximately 20 min.

T2 measurements were performed in a 3T MRI scanner (MR750W, GE Healthcare, Waukesha, WI) using a double-echo fast spin-echo (FSE) sequence with and without water suppression (echo time (TE) = 35/182 ms and 28/147 ms, repetition time (TR) = 1500 ms, echo train length (ETL) = 40, field of view (FOV) = 12 cm, 128 × 128 matrix size, 8-mm slice thickness, 15 s acquisition time). Water suppression was achieved via a frequency selective pulse centered on the water frequency. To ensure consistency, the frequency of the fat peak was verified before each scan, and the same pre-scan parameter values were used throughout the experiment. Images were acquired during heating (25, 35, 45, 55, 65, and 70 °C) and subsequent cooling (55, 35, and 25 °C) after reaching thermal equilibrium. T2 maps were generated with an exponential fit for two data points.

### Trabecular bone marrow ablation

Two ex vivo experiments were performed on epiphysis segments of bovine femur with a birdcage head coil (GE Healthcare) at 3T. The samples were cut to expose the trabecular tissue containing red marrow to the ultrasound beam. Three fiber optic sensors were placed into drilled holes within the red marrow. The femoral segments were sonicated with the conformal bone focused ultrasound system (ExAblate 2100, InSightec, Israel) operating at 500 kHz, acoustic power of 17.6 W, and a sonication time of 8 min, with the focus placed within the bone marrow. T2 was quantified by using the double-echo FSE sequence with water suppression described above (TE = 35/181 ms, TR = 723 ms, echo train length = 40, FOV = 24 cm, 128 × 128 matrix size, 10-mm slice thickness, 15 s/slice). Images were acquired during heating and cooling and after the sample reached room temperature. T2 values were measured within three elliptical regions of interest (ROIs) (area: 8.6 mm^2^) placed over the locations of the tips of the fiber optic sensors.

### Intact cortical bone ablation

In order to test T2-based temperature monitoring in conditions more closely resembling those of a bone treatment, we performed ablation in an intact ex vivo porcine femur (sonication time: 20 s, acoustic power: 30 W) using the in-table transducer of the ExAblate system, operating at 1.15 MHz. The focus of the sonication was placed within the yellow marrow of the diaphysis of the femur with the ultrasound beam path intersecting the cortical bone. Bone marrow T2 was quantified with a double-echo FSE sequence with water suppression (TE = 35/186 ms, TR = 1500 ms, echo train length = 40, FOV = 32 cm, 128 × 128 matrix size, 10-mm slice thickness, 15 s/slice).

### In vivo temperature monitoring

**Fig. 1:**
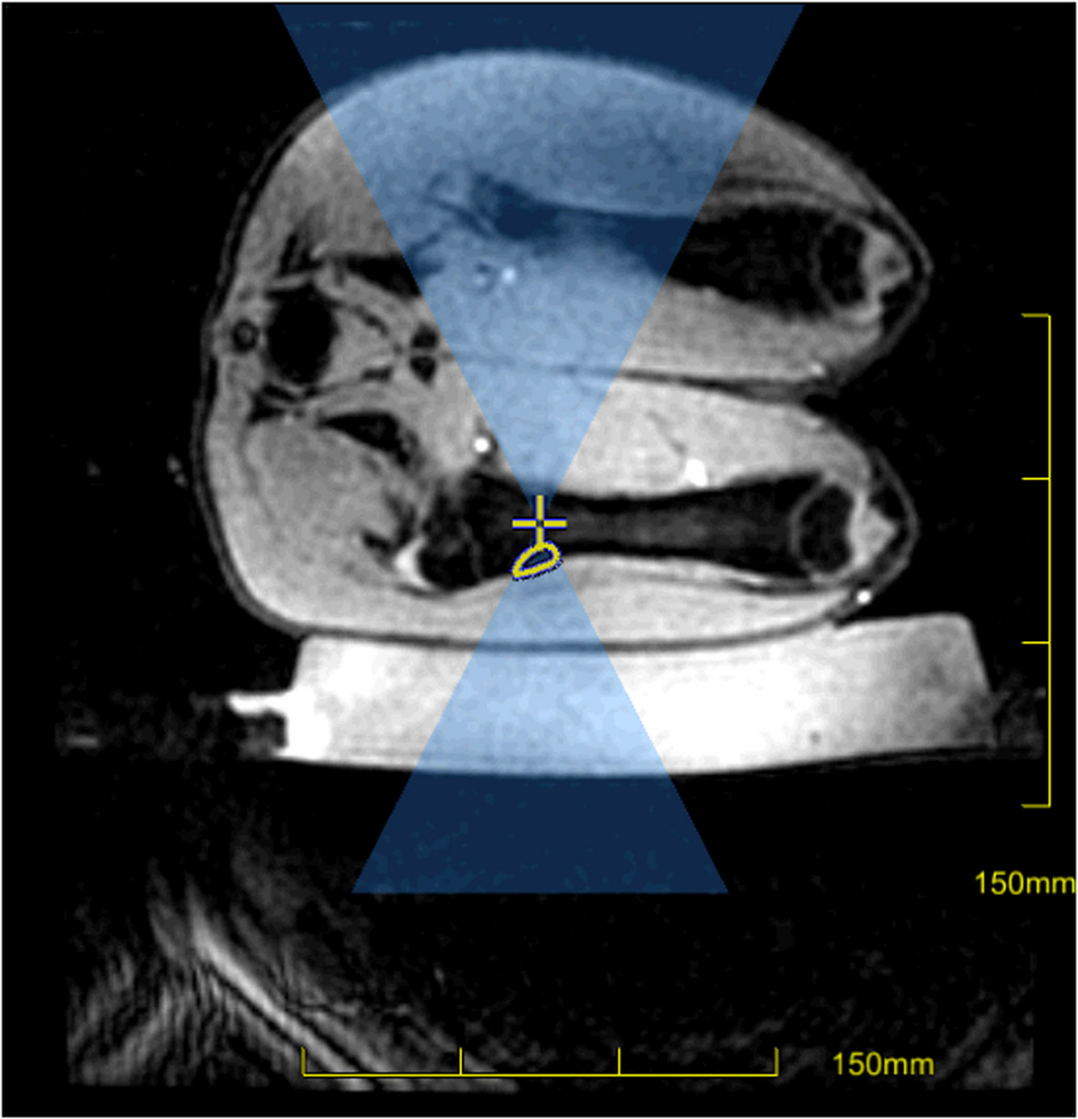
Planning image of one of the sonications in a swine model with the path of the US beam overlaid in *blue*, the focal point as a *yellow cross*, and the area of expected thermal dose as a *yellow outline*

Focused ultrasound ablation was also performed in a swine model. All experimental procedures were done in accordance with National Institutes of Health (NIH) guidelines for humane handling of animals and received prior approval from the University of California San Francisco Institutional Animal Care and Use Committee (approval number: AN088193). The animal was positioned in the left decubitus position onto a gel pad over the ExAblate in-table transducer (Fig. 1) using a frequency of 1 MHz. A total of 12 sonications were performed on the distal diaphysis (six sonications, 35 W, 20 s) and proximal diaphysis (six sonications, 17 W, 40 s) of the femur. As in the ex vivo validation, the focus was placed inside the medullary cavity. At the end of the focused ultrasound procedure, pre- and post-contrast 3D fast spoiled gradient echo (SPGR) images were acquired.

## Results

### Yellow bone marrow temperature calibration

**Fig. 2:**
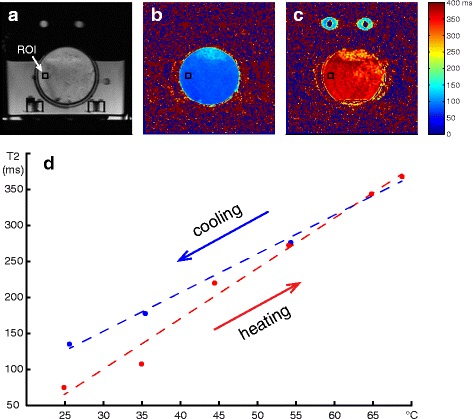
FSE images of a petri dish (no water suppression), containing bovine yellow bone marrow within the insulated water bath (**a**); T2 maps of the sample at 25 °C (**b**); and at 70 °C (**c**); plot of T2 versus temperature for ex vivo bone marrow during heating (*red*) and cooling (*blue*) (**d**) acquired with TE = 35/182 and water suppression

Figure 2 shows examples of T2 maps of ex vivo bone marrow at 25 and 70 °C acquired with TE = 35/182 ms and water suppression. The T2 values within a 10 × 10 pixel ROI (black square on Fig. 2a) versus the temperature of the water bath at equilibrium are plotted in Fig. 2d. The linear regression line of the data acquired during heating exhibited a steeper slope than that of the cooling phase.

Table 1 shows the linear regression coefficients of T2 versus temperature for the different acquisition parameters. There was approximately a 25 % difference between the measurements with and without water suppression. The difference in the T2/temperature coefficients between the two sets of echo times was smaller.

**Table 1:** Linear regression coefficients of T2 versus temperature (ms/°C) for the different pulse sequence parameters

	Heating	Cooling
Water Suppr. TE = 28/147	7.05	5.39
Water Suppr. TE = 35/182	7.00	5.39
No Water Suppr. TE = 35/182	5.48	4.47

### Trabecular bone marrow ablation

**Fig. 3:**
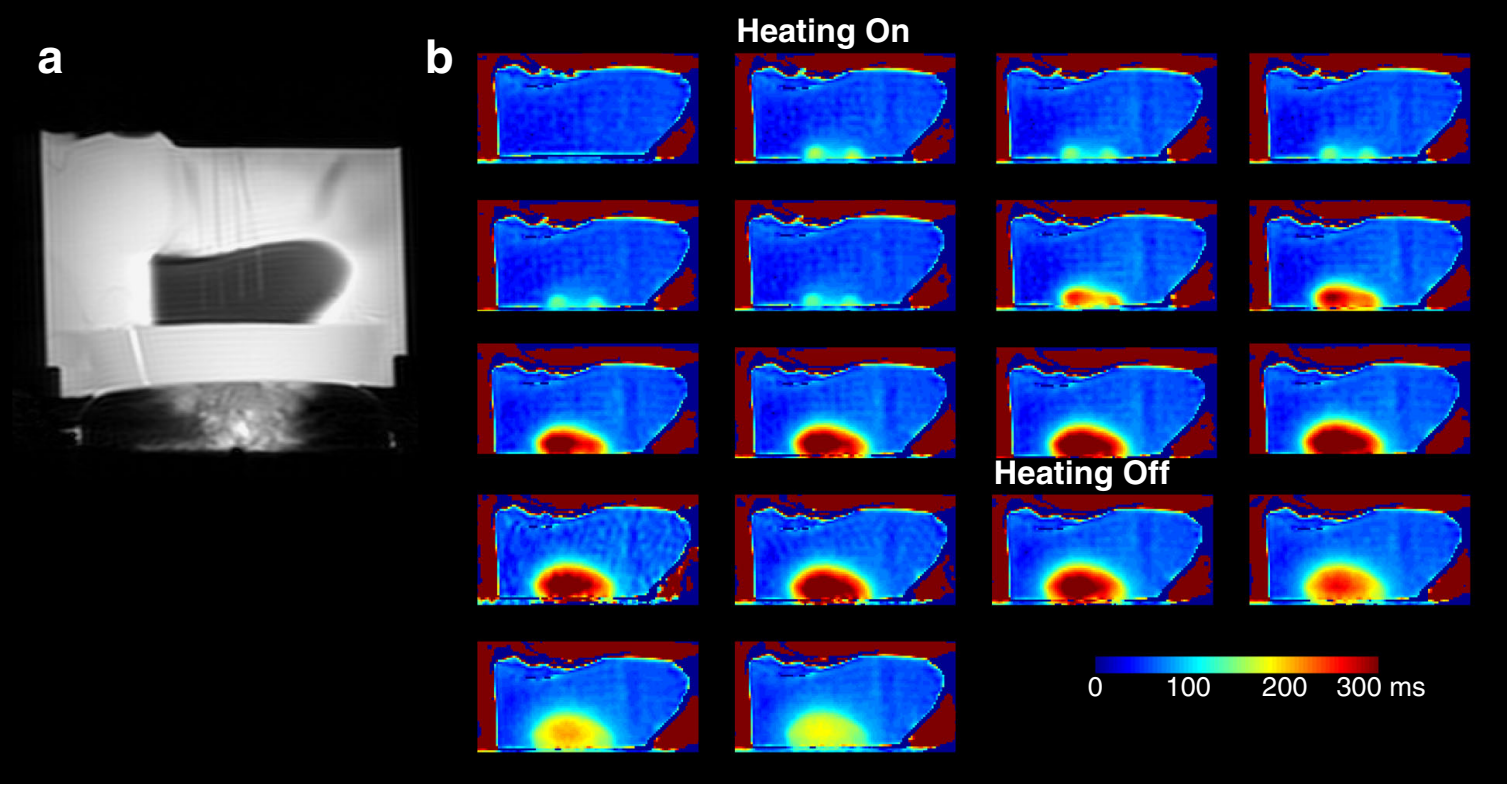
**a** Magnitude image acquired during sonication showing the setup for T2-based thermometry of trabecular bone marrow during focused ultrasound sonications showing the fiber optic probes; **b** T2 maps of sample 1 during heating and cool-down

Figure 3 shows the T2 change during heating and cooling of a sample of trabecular bone marrow. As can be seen, despite the focal spot being deep within trabecular bone, most of the energy was absorbed at the edge of the sample and then radiated into the marrow.

**Fig. 4:**
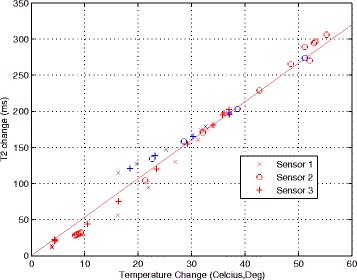
Temperature change, measured by three probes within the bone marrow sample and T2 change at the same locations during heating (*red*) and cooling (*blue*)

Figure 4 shows a plot of T2 change versus the temperatures over time, measured by the three fiber optic probes. There was a linear relationship (5.7 ms per °C) during the heating stage of the experiment (red points). The T2 change during cooling (blue) showed a reduction with temperature, but did not follow the same linearity as during heating.

**Fig. 5:**
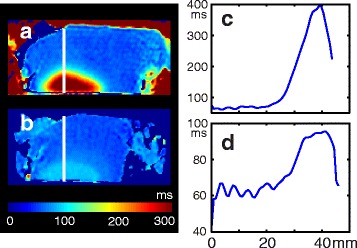
T2 maps of sample 2: **a** during heating; **b** after reaching room temperature; **c** T2 profile of the sample along the line, shown in **a**; **d** T2 profile along the line, shown in **b**

Figure 5 shows the T2 change of another bone segment during heating (a) and after it returned to room temperature (b). The profiles through the heated region (Fig. 5c, d) show residual T2 elevation of about 35 ms in the ablated area suggesting irreversible tissue changes. The area of residual T2 elevation after cooldown matched the area of the heating.

### Intact cortical bone ablation

**Fig. 6:**
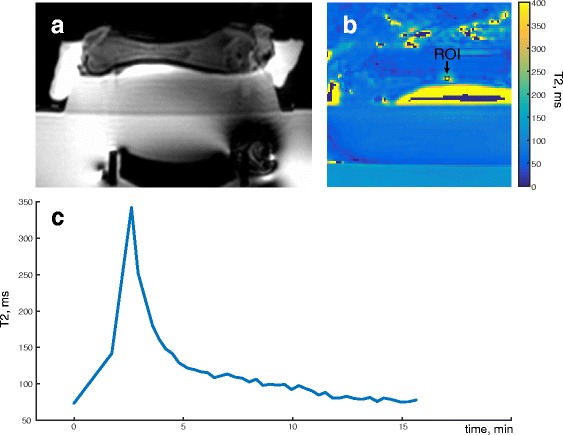
T2 measurement in ex vivo bone marrow during and after the heating. **a** Localizer image showing the ultrasound transducer in the table. **b** T2 map during heating, showing the ROI. **c** Plot of T2 values within the ROI over time

Figure 6 shows the results of the ex vivo experiment, where we measured a T2 elevation of 269 ms. We observed an increase in T2 values in the area of the marrow adjacent to the intersection of the cortical bone and the beam path. Assuming a linear coefficient of 7 ms/°C from the yellow marrow calibration experiment, this corresponded to a temperature rise of 38 °C. The ex vivo experiment showed that it took on the order of 15 min for the marrow to return to baseline temperature.

### In vivo temperature monitoring

**Fig. 7:**
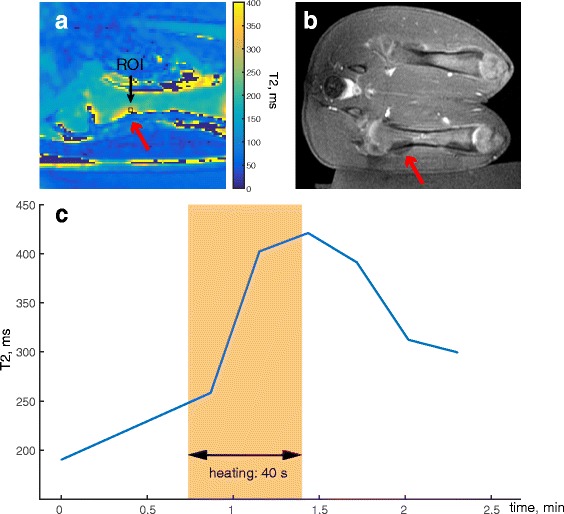
T2 measurement in in vivo bone marrow: **a** T2 map during ablation of a single sonication, showing the ROI; **b** post-contrast 3D Fast SPGR image after ablation (total of six sonications per location); **c** plot of T2 values within the ROI over time; the *highlighted area* shows the approximate duration of the sonication

Figure 7 shows the results of one of the sonications of the in vivo experiment in a swine model. An area of elevated T2 values in the marrow, adjacent to the cortical bone in the US beam path appeared on the T2 maps acquired during heating (Fig. 7a). From the plot of the T2 values within an ROI over time (Fig. 7c), we observed that the highest T2 values appeared at the end of the sonication. We measured a T2 rise of 231 ms within the bone marrow, which corresponded to temperature change of 33 °C from baseline according to the data from the calibration experiment. The in vivo experiment showed excellent correspondence between the area of T2 elevation in marrow during the ablation and the resulting non-enhancing area in the post-contrast images (Fig. 7b).

## Discussion

In this study, we have measured the temperature dependency of T2 in yellow and red bone marrow, developed T2-based thermometry for monitoring of MRgFUS ablation of trabecular and cortical bone, and validated the technique in an animal experiment.

First, we have calibrated the temperature dependence of T2 in yellow bone marrow for temperatures between 25 and 70 °C at 3T. We found that sequence parameters influence the relationship between the measured T2 values and temperature. In particular, there was a large difference between the measurements with and without water suppression. Although yellow marrow mainly consists of fat cells, there could still be a contribution of water spins to the measured T2 in the non-water-suppressed acquisitions or a portion of the fat spins could be suppressed in the water-suppressed acquisition. Changing the echo times had less effect on the measured values. In order to achieve high temperature accuracy, the calibration of T2-based thermometry techniques should be done with the same parameters as those used for temperature monitoring during the treatment of patients. To maintain consistency despite varying concentration of water in the marrow, water suppression should be used for T2-based thermometry of fatty tissues and was employed in the rest of the experiments presented here.

In the experiments in ex vivo trabecular and cortical bone, we observed that even though the focal points were placed in the middle of the bone, due to the high ultrasound absorption of bone, most of the energy was absorbed at its edge. The trabecular bone ablation experiments, where heating continued for 8 min, showed that there was a residual change of T2 values in the area of the marrow that experienced heating even after the sample returned to the room temperature. This was consistent with the results of our calibration experiments in yellow and red marrow, where T2 values followed a more gradual slope on the cooldown from high temperatures then the values observed during heating. Similar effect has been previously observed in subcutaneous fat after exposure to high temperatures [23]. Elevated T2 values in the areas of thermal damage could be an indicator of irreversible tissue changes and allow for evaluation of treatment effects during bone MRgFUS therapy. We did not observe residual T2 changes in the marrow of the ex vivo or in vivo cortical bone ablation. Either the shorter sonication duration did not cause irreversible changes or changes were not apparent due to lower spatial resolution.

In our animal experiment, we observed a T2 increase in the marrow of 231 ms from the baseline value of approximately 200 ms during heating. Baseline values in the ex vivo samples were much lower due to the difference in initial temperature. The area of elevated T2 in the marrow matched the non-perfused volume in the post-contrast images. The highest temperature in the marrow was achieved very close to the end of the sonication. This is contrary to PRF measurements in muscle adjacent to the cortical bone, where the maximum temperature is reached 10–15 s after the end of the sonication [16]. This suggests that temperature in marrow could be a more direct measure of bone heating than the temperature in surrounding muscle tissues. Monitoring temperature in marrow could reduce the risk of overtreatment due to underestimation of the temperature of the bone.

As a proof of concept work, this study had several limitations. The T2-based thermometry technique was performed in a very small number of samples and animals to date. Future work is needed to validate the technique in a larger population. For the ex vivo experiments, we used previously refrigerated samples. There may be a difference between T2/temperature coefficients between refrigerated, freshly excised, and in vivo tissue, as well as between bovine, porcine, and human bone marrow. In vivo and ex vivo cortical bone ablation experiments relied on the previously estimated calibration coefficients, since it would be very difficult to embed temperature sensors in those samples and animals, and precisely register the sensor tip to the corresponding voxel in an area of large temperature gradients. In our calibration experiments we used yellow marrow, which was extracted from the medullary cavity. In humans or animals, the medullary cavity may contain either yellow, red, or, not uncommonly, a blend of both types of marrow. The effect of the marrow composition on the temperature coefficient remains to be investigated. The T2/temperature calibration in this work has been performed at 3T. The coefficient is likely dependent on field strength and should be calibrated with the same acquisition parameters that would be used in vivo.

The technique itself has several limitations at this stage. Achieving consistent water suppression was difficult since we used frequency selective suppression, which was very sensitive to pre-scan parameters, as well as B0 and B1 inhomogeneities. Other techniques such as spectral spatial excitation and inversion recovery could be considered for suppression of the water signal. It is currently not possible to simultaneously acquire both T2-based thermometry in the marrow and PRF-based thermometry in the surrounding muscle, since those techniques use different pulse sequences. This could be accomplished with interleaved acquisition techniques [25]. The technique described here supported a single slice imaging with 15 s acquisition time. Accelerated k-space readout trajectories, such as echo-planar or spiral, could improve spatial coverage and temporal resolution of the technique.

## Conclusions

We have shown that T2-based ablation monitoring in red and yellow bone marrow is feasible. Our results were consistent with previously published data in marrow and subcutaneous fat. The linear relationship between T2 change and temperature could be used to quantify the temperature during heating of up to 60 °C. This would allow to reliably and accurately monitor the temperature inside the trabecular bone during treatment of patients with bone tumors. The ability to monitor the temperature within the bone marrow allowed more complete visualization of the heat distribution into the bone, which is important for local lesion control and treatment of osteoid osteomas. Therefore, T2-based temperature mapping, in addition to PRF-based thermometry, could be used to monitor heating during the bone focused ultrasound treatments and improve safety and efficacy of MRgFUS bone applications.

## Abbreviations

ETL: Echo train length
FDA: Food and Drug Administration
FOV: Field of view
FUS: Focused ultrasound
MRgFUS: MR-guided focused ultrasound
NIH: National Institutes of Health
PRF: Proton resonance frequency
ROI: Region of interest
SNR: Signal-to-noise ratio
SPGR: Spoiled gradient echo
TE: Echo time
TR: Repetition time
US: Ultrasound
UTE: Ultrashort echo time
